# Airborne Rodent Allergen Levels in Dutch Households: A Pilot Study

**DOI:** 10.3390/ijerph16193736

**Published:** 2019-10-04

**Authors:** Sara A. Burt, Lidia I. Parramon Dolcet, Inge M. Wouters

**Affiliations:** Institute for Risk Assessment Sciences, Faculty of Veterinary Medicine, Utrecht University, P.O. Box 80178, 3508 TD Utrecht, The Netherlands

**Keywords:** rat, mouse, asthma, indoor exposure, Mus m 1, Rat n 1, airborne dust, home

## Abstract

Little research has been conducted in Europe regarding indoor exposure to airborne rodent allergens. The aims of this study were to gain insight into the prevalence of rodent allergens in airborne dust in Dutch households, to assess whether there is a relationship between rodent sightings and detectable allergens, and to identify risk factors associated with the presence of rodent allergens. Airborne dust was collected from the living rooms of 80 households distributed around central Netherlands by passive sampling using electrostatic dust collectors (EDCs). Samples were analysed for mouse (Mus m 1) and rat (Rat n 1) allergens. Participants completed a questionnaire on household and building characteristics, household pets, cleaning habits and ventilation. Mouse allergen was more prevalent than rat allergen and mouse sightings within the past year more than doubled the odds of detectable mouse allergen. Proximity to green areas, ventilation through an open window and insulation under the living room floor were determinants for detectable mouse allergen. Conversely, proximity to surface water was protective. No significant association was found between asthma and detectable mouse allergen. The passive EDC sampling method was used successfully to detect mouse and rat allergens in homes. The presence of mouse allergen was associated with previous mouse sightings. Risk factors and protective factors associated with the presence and levels of mouse allergen were identified.

## 1. Introduction 

Allergens of mice and rats in the home have been associated with asthma and asthma exacerbation, especially among sensitized children [1,2,3]. Research conducted in the USA reports high levels of rodent and cockroach allergens in inner-city homes [2,4]. In a nationwide study conducted in 831 homes, mouse indoor allergen exposure was estimated by testing dust samples from furniture and the floor. Eighty two per cent of homes had detectable levels of mouse allergen and a third of homes had levels high enough to increase the risk for occupants of developing allergies (1.6 µg/g mouse urinary protein) [5]. Other studies have confirmed that exposure to mouse allergen in US homes is quite common and that residential allergen burden is strongly affected by sociodemographic factors [1,4,6,7]. Exposed food remains, cracks or holes in the walls, and evidence of mouse infestation appear to be the main risk factors for having detectable airborne mouse allergen in the house [8]. Other predictors associated with the detection of mouse allergen are the size of the home, pesticide use, having a private garden, frequent window opening, and the presence of pets in the home [6,9].

Few studies have evaluated indoor exposure to rodent allergens in Europe [10]. A French study of 60 randomly selected homes found mouse allergen in 36 (60%) of them and rat allergen in none [11]. A Dutch study detected mouse urinary protein in airborne dust from schools and homes [12]. However, the analysis of determinants was limited. In a recent online survey in the Netherlands, just over 60% of the respondents reported sightings of rodents in or around the house during the previous year [13]. Even allowing for possible selection bias in that survey, it would appear that a significant proportion of homes are host to rodent infestations, in which case the inhabitants could be exposed to airborne rodent allergens. We therefore proposed to investigate the presence of mouse and rat allergens in homes distributed around the central Netherlands. 

Acquiring dust samples is mostly done by vacuuming floors or furniture and some studies have handed over electrostatic dustfall collectors (EDCs) to participants. We wished to test whether recruitment by flyering and sending out EDCs to participants by post are feasible to conduct a larger study later on.

The aims of this study were (i) to gain insight into the prevalence of mouse and rat allergens in Dutch households, (ii) to assess whether there is a relationship between rodent sightings and detectable allergens in the home, and (iii) to identify risk factors associated with the presence (and levels) of rodent allergens in the home. 

## 2. Materials & Methods

### 2.1. Study Design

This cross-sectional study aimed to gather a broad range of households with different ages of property covering the central area of the Netherlands. Participants were invited to take part in a study considering household allergen exposure, without specifically referring to rodent allergens. Most invitations were distributed by hand to homes in different areas of the city of Utrecht, in addition four pest control companies and two local health authorities contributed to the distribution of invitations to householders to take part in the study. People willing to participate in the study received a questionnaire and an electrostatic dustfall collector (EDC) with instructions on how to use it. Participants were asked to deploy the EDC for four weeks, to complete the questionnaire and to return both items via the post in the envelope provided. A contact telephone number was provided in case of any questions. The EDCs were analysed for the presence of mice and rat allergens using an ELISA assay and the data was related to the answers in the questionnaire.

Given that airborne rodent allergen levels were expected to be low, optimization of deployment time was explored prior to handing out the invitations to take part in the study. To this end, EDCs were deployed for 2, 4, 6 and 8 weeks in a laboratory animal facility containing rodents, in the canteen of the facility, and in two offices in the same building but not near the animal facility. Rodent allergen content was determined and associations with deployment time were investigated.

### 2.2. Recruitment of Study Participants

An invitation letter was delivered to about 600 homes in Utrecht and other towns in the Netherlands in April–May 2017. The neighbourhoods were selected in order to represent different housing characteristics (age of buildings, economic status, proximity to canals or green areas, etc.). We distributed invitations to every house on 2–3 streets that were characteristic for the different neighbourhoods. Two local area health authorities also handed out about 45 invitations and four pest control companies operating nationally also distributed about 50 invitations. Eighty households took part in the study between April and August 2017.

### 2.3. Patient and Public Involvement

The study was an open population study and therefore no specific patient representatives were involved in the set-up and design of the study. Participants from the general population, not patients, were involved in the execution of the study. All participants were provided with a written report on the outcomes of the study.

### 2.4. Questionnaire

The questionnaire included questions related to topics previously reported in the literature to be associated with the presence of rodent infestations in the house, such as household characteristics, health of the participant, ventilation of the house, socioeconomic status, presence of pets in the house and smoking. The main purpose of the questionnaire was to identify risk factors or determinants associated with the detection of rodent allergens indoors.

### 2.5. Collection of Airborne Dust Samples 

Airborne dust was collected over a four-week period in each home by passive sampling. An electrostatic dustfall collector (EDC), first described by Noss [14], was sent out to participants, who were instructed to lay it on a cupboard or bookcase at a height of at least 1.25 m above floor level in the main living room. The EDCs were returned to the laboratory by post and were stored at −20 °C until analysis. The Dutch postal system delivers letters and packages within 24 h on 5 days a week, so the maximum delivery period was 3 days (https://www.postnl.nl/zakelijke-oplossingen/post-versturen/postbezorging-en-aanlevering/). The EDCs were stored at −20 °C immediately upon arrival in the lab until further processing to inhibit the activity of dust mites which may have been collected on the EDCs. Dust mites live in house dust and consume dust particles. By consuming dust, they could reduce the amount of rodent allergens in the samples before they could be extracted and analysed. The span of 1–3 days during postal transit was deemed acceptable because it is relatively small compared to the period of several weeks or months between arrival of the material from the first and the last participants in the study. 

The EDCs were returned in a closed plastic holder in a sealed plastic bag inside a sealed envelope and therefore posed negligible health risk to postal workers (less than if they were to enter one of the houses in question). 

### 2.6. Allergen Extraction

After thawing to room temperature, allergens were extracted by incubating each EDC in a tube containing 20 mL of phosphate buffered saline with 0.05% v/v Tween-20 in an end-over-end roller for 60 min at room temperature. EDCs were transferred using tweezers to handle the EDCs whilst wearing protective clothing in a lab with normal air circulation, minimizing health risks to lab personnel. Two mL of each sample were transferred to a fresh tube and centrifuged for 15 min at 2000× *g*. Aliquots of the supernatant were stored at −20 °C until further analysis.

### 2.7. Measurement of Allergen Levels

Each sample was assayed for mouse and rat urinary proteins Mus m 1 and Rat n 1 using commercial ELISA kits (Indoor Biotechnologies, Inc., Cardiff, UK) following the manufacturer’s instructions with slight adaptations to improve sensitivity. Adaptations included using streptavidin with poly horseradish peroxidase (HRP) to improve immunoassays, as first described by Korpi et al. [15]. Briefly, 96-wells plates (Greiner BioOne BV, Alphen aan den Rijn, The Netherlands) were coated overnight with 100 µL of a 1:1000 diluted catching antibody in coating buffer. The next day, samples were added in 1:2 serial dilution, and the Universal Allergen Standard curve consisting of 8 dilution points in duplicate were added, starting with a concentration of 62.5 pg/mL for mouse allergens and 625 pg/mL for rat allergens. Next, detection antibodies were added according to manufacturer dilutions, followed by streptavidin poly-HRP (Sanquin, Amsterdam, The Netherlands) in a dilution of 1:40,000. O-phenylenediamine dihydrochloride (100 μL/well) was used as a substrate for HRP and incubated for 30 min at room temperature. The colour reaction was stopped by adding 50 µl of 2 M HCl-solution. The colour reaction was read at 492 nm wavelength by ELISA plate reader (Versamax, Molecular Devices, San Jose, CA, USA).

### 2.8. Statistical Analysis

Statistical analyses were performed using SPSS version 24 (IBM Corp., Armonk, NY, USA). Univariate and multiple logistic regressions were performed to investigate determinants associated with the presence of detectable mouse and rat allergens in the home. A linear regression model was used to find out which variables were associated with log-transformed level of mouse allergen in the home. In cases where allergen was under the limit of detection, a value of two thirds of the limit of detection was assigned. Only data for mouse allergen were used to perform logistic and linear regressions as rat allergen was detectable in too few households to make statistical analysis feasible.

## 3. Results

### 3.1. Prior Determination of Duration of Sampling Period

**Figure 1:**
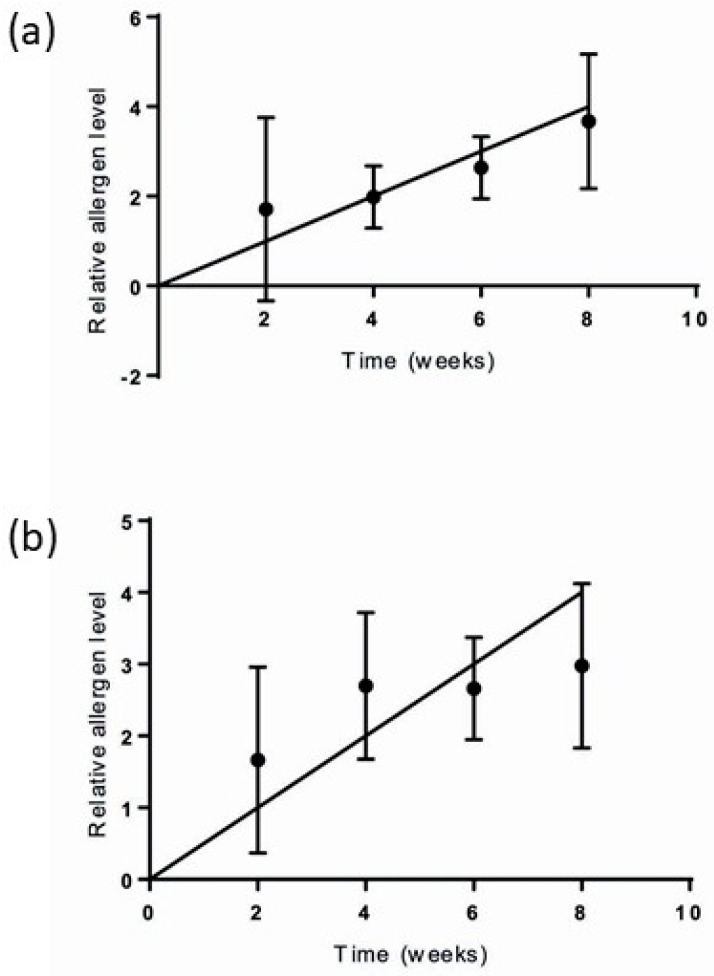
Relative allergen level increase in electrostatic dustfall collectors (EDC) with increasing sampling duration: (**a**) mouse allergen; (**b**), rat allergen. The data points show relative allergen level. The bars show standard deviation.

Given that airborne rodent allergen levels are expected to be low, optimization of deployment time was explored prior to handing out the invitations. To this end, EDCs were deployed for 2, 4, 6 and 8 weeks in a laboratory animal facility containing rodents, in the canteen of the facility, and in two offices in the same building but not near the animal facility. Rodent allergen content was determined as below, and associations with deployment time were investigated (see Figure 1). Based on the results, a sampling duration of 4 weeks was chosen.

### 3.2. Participants and Household Characteristics

The demographic information on the study participants is presented in Table 1. The characteristics (age, sex, smoking habits, etc.) of the person who filled in the questionnaire were requested, not the characteristics of other family members. Demographics of the participants showed women to be more likely to participate than men, and people of middle age category and higher education participate slightly more than other groups. This is not uncommon for this type of studies. Data in our study are largely comparable to demographics in other studies of Dutch households [13,16].

In total, 80 households took part and most lived in the city of Utrecht (78.8%). The geographical distribution of participants is shown on a map in Supplementary Materials. Forty percent of participants reported one or more mouse sightings during the last year and ten percent reported rat sightings. The geometric mean of the level of allergens in settled dust in positive houses was 2.5 ng/m^2^ for mouse allergens (GSD 3.6) and 39.3 ng/m^2^ for rat allergens (GSD 3.4).

Descriptive information on housing characteristics and cleaning habits is presented in Table 2. The majority of participants lived in terraced houses (63.8%) and had a private garden (85.0%). 

### 3.3. Risk Factors Associated with Detectable Allergens in the House

The univariate logistic regression analysis (see Table 3) showed that having a pet cat significantly reduced the odds for detectable mouse allergen in the home (*p* < 0.05). In contrast, ventilating the living room by means of an open window increased the odds of detecting mouse allergen. Some other characteristics like mouse sightings in the last 12 months, living in a house built in 1920–1960, and living close (less than 250 m) to a green area showed a trend for association with detection of mouse allergen (*p* < 0.10).

The results of the multivariate logistic regression analysis for detectable mouse allergen are presented in Table 3. Living close (less than 250 m) to a green area increased the odds of having detectable mouse allergen but living less than 250 m from surface water reduced the odds (*p* < 0.05). Having the floor of the living room insulated or ventilation of the living room through an open window increased the odds of having detectable mouse allergen. With a more lenient restriction on the p-value (*p* < 0.1), mouse sightings tended to increase the odds of having detectable mouse allergen and the use of furniture polish reduced the odds of having detectable mouse allergen in the house.

In sensitivity analyses, restricting the population to households in Utrecht did not majorly affect the factors and direction of associations observed in the total population (results presented in Table 3).

### 3.4. Risk Factors Associated with Higher or Lower Allergen Levels

Linear regression was carried out to identify characteristics associated with higher or lower airborne levels of mouse allergen and the results are presented in Table 4. The univariate analysis showed that dusting at least once a week significantly reduced levels of allergens (*p* < 0.05). The multivariate analysis showed that living close (less than 250 m) to a green area and having an insulated floor increased the levels of mouse allergen. On the other hand, living close to surface water, and the use of furniture polish and/or multipurpose cleaner reduced allergen levels. 

In the sensitivity analyses restricting the population to households in Utrecht only, some other factors were associated with mouse allergen levels, although most of the characteristics observed in the total population remained (results presented in Table 4).

### 3.5. Relation between Sightings and Detectable Allergens

**Figure 2:**
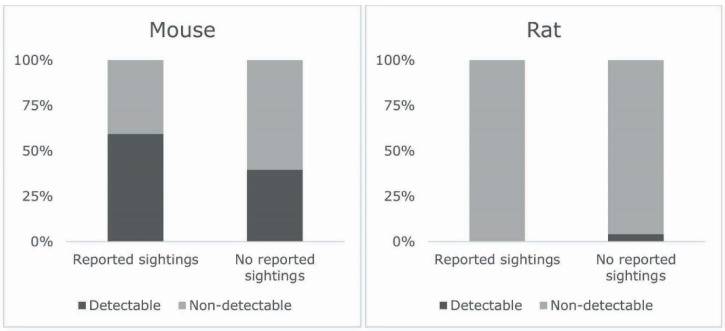
Prevalence of detectable allergens in study participants’ homes stratified by reported mouse or rat sightings.

Mouse allergen was detectable in 47.5% of the homes and rat allergen in 3.8%. Figure 2 depicts the percentage of households with detectable and non-detectable level of allergens stratified by reported corresponding rodent sightings. Participants who reported mouse sightings were more than twice as likely to have mouse detectable allergen in the house than people who did not report sightings (univariable OR = 2.2; 95% CI, 0.90–5.55). However, more than a third of the people who did not report mouse sightings also had detectable mouse allergen in the home. None of the homes where rat allergens were detected had reported sighting a rat during the past year.

### 3.6. Relation between Allergens and Asthma

Less than 12% of the people who participated in the study suffered from asthma. No significant association was found between self-reported asthma and having detectable mouse allergen levels in the house (OR = 1.44; 95% CI, 0.22–3.71).

## 4. Discussion

This is the first study to collect airborne rodent allergens in the living rooms of Dutch homes and to identify building characteristics and cleaning habits as risk factors for the presence and level of mouse allergen. Mouse allergen was found in almost half of 80 homes and rat allergen in just under 4%. Risk factors for the presence of mouse allergen were proximity to a green area, ventilation by means of an open window, and having an insulated living room floor. In contrast, proximity to surface water and the use of furniture polish were protective. Levels of mouse allergen were influenced by the same factors and by use of a multipurpose cleaner (associated with lower levels). No significant association was found between self-reported asthma and detectable mouse allergen. 

Our findings on the frequency of measurable rodent allergens in homes are comparable with the two previous European studies. In a study of inner-city homes in Poland, 46% of 39 dwellings contained detectable mouse allergen (rat allergen was not examined) [17] and in a French study 60% of 60 dwellings had measurable concentrations of mouse allergen and rat allergen was not detected [11]. A Mexican study of 264 homes also detected Mus m 1 in 60% of them and Rat n 1 in 10% [9]. The frequencies of measurable mouse allergen in the USA are generally higher; in a national survey, Mus m 1 was detected in 82% of homes [4]. 

The method we used to collect airborne dust was different to most studies, which collected dust by vacuuming dust from floors or furnishings [4,9,11,17]. The collection of airborne dust by EDC should provide a more realistic estimate of exposure to allergens via inhalation because dust and dirt brought in on the feet of inhabitants and their pets would also be collected by vacuuming but would not be collected by EDCs. However, it precludes a direct comparison to earlier reported allergen levels. 

We conclude that this method of acquiring dustfall samples is suitable and convenient for use with the public, although if participants are recruited by post the response rate will be low. The EDCs are easy to distribute and collect by post and it is easy for the public to follow the instructions for use. A larger scale study is being planned in order to provide more precise assessment of presence and levels of rodent allergens in Dutch homes and to enable analysis for possible effects on asthma.

### 4.1. Demographics

This study found no significant association between socioeconomic status (SES), assessed as the level of education of the participants, and the presence of allergens. This may be because the majority of participants (83.3%) had obtained a high level of education. An earlier Dutch study looking at allergens in children’s bedrooms and schools also found that the SES was not significantly associated with mouse allergen [12]. In contrast, a study in the USA found that a higher concentration of mouse allergen in the home was associated with lower income [4,5].

The earlier reported association between smoking in the home and a higher percentage of households with high mouse allergen levels [5,18], was not confirmed in this study. The lack of an association may be due to almost 90% of the participants being non-smokers or rarely smoking in the house. 

### 4.2. Building Location and Characteristics

A number of determinants was associated with the presence of mouse allergen. A number of these have been reported by earlier authors and others have not. 

Living close to a green area was found to increase the odds for detectable mouse allergen in the home. Although house mice normally inhabit buildings, wood mice usually live in gardens and parks and may enter premises when foraging for food [19,20]. However, wood mice do not excrete Mus m 1 and so should not contribute to the allergen load measured in this study (Pavel Stopka, personal communication). A study on rats in the city of Amsterdam evaluated the association between rat sightings and the presence of vegetation. Rat sightings were increased in green areas, suggesting that rodents may have a preference for areas with vegetation within urban areas [21]. A recent study on mouse allergens in Chicago bedrooms found that the presence of Mus m 1 was correlated with flowering plants in the neighbourhood, but not with having trees nearby [6].

Living close to surface water was protective against mouse allergen. Mice are not particularly attracted to water. In fact, depending on their diet, house mice can survive without water [19]. This is in contrast to brown rats, which prefer habitats close to water [21].

Ventilation through an open window increased the odds for having detectable mouse allergen. An earlier study considered ventilation through an open window as a potential predictor for mouse allergen in the home, although no significant association was found [9]. A more recent study found a significant correlation between frequent window opening and several allergens including Mus m 1, suggesting that outside air, or increased turbulence, contributes significantly to the burden of mouse allergen in homes. This phenomenon may contribute to allergen burden in the absence of rodent infestation [6]. Without having sampled the outside air, we cannot confirm this source of allergens in the present study. In future studies, asking people to record the duration of window opening or recording the airflow in the room where the EDC is placed would improve precision.

Unexpectedly, insulation of the living room floor was found to be significantly associated with an increase of mouse allergen detection in the house. No previous reference to an association between mouse allergen and insulated floors could be found in the literature. We hypothesise that houses with floor insulation are more likely to be generally better insulated all round and, therefore, possibly less well ventilated than uninsulated houses. Better general ventilation may lead to a reduction in mouse allergen in the air. Another hypothesis may be that the insulation may attract mice to settle, although this is highly speculative.

In a subset of homes, namely those situated in Utrecht, buildings built before 1920 were found to be significantly associated with increased levels of mouse allergen. This finding is in accordance with other studies that found that older homes were associated with increased concentrations of allergens or were more likely to contain mouse allergens than younger houses [4]. In Amsterdam, houses built before 1960 are associated with a higher percentage of rat reports than younger homes [21]. It is possible that older buildings are more sensitive to maintenance problems and the use of building materials that can be gnawed by rodents, such as wood, which may contribute to this. 

Having a private garden was not a statistically significant determinant for detectable mouse allergen in the home. This finding differs from studies that concluded that houses with a garden or with flowering plants nearby had increased chances of having detectable mouse allergen [6,9].

### 4.3. Household Cleaning Habits 

We found that the use of furniture polish was protective against detectable mouse allergen and there was a significant association between the use of furniture polish or multipurpose cleaner and lower levels of mouse allergen. In the univariate analysis, dusting at least once a week was also associated with lower levels of allergens (Table 4). These findings concur with studies that found extensive cleaning to be a successful intervention to reduce rodent allergens in homes [22,23]. The use of certain chemicals such as pesticides or insecticides was earlier found to be protective for rodent and other pest allergens. On the other hand, perfumed products to clean the house were found to be a risk factor [9]. 

Having a smell of mildew or mould in the house tended towards a low level of allergen, although this was only statistically significant for homes in the city of Utrecht. In contrast, in earlier studies cracks or holes in the walls and moisture problems have been found to be predictors of high mouse allergen levels [5,8,18]. It is possible that noticing the smell of mildew stimulates people to clean their home more often, which may reduce the presence of allergens.

### 4.4. Relation between Mouse Sightings and Allergen Levels

Mouse sightings tended to be associated with the detection of mouse allergen (OR = 2.2). This finding concurs with other studies that found that having problems with mice in the previous year is a predictor for detectable allergen [2,8,9]. However, almost one third of participants who did not report mouse sightings did have detectable mouse allergen in the home. This finding supports work carried out in Los Angeles, where half of the homes that did not report rodents had detectable mouse allergen [18]. Possibly, mouse allergen entering through open windows may contribute to levels in homes with no rodent infestation [6]. Previously, we found that approximately 16% of mice caught during pest control operations in buildings are wood mice (Apodemus sylvaticus) [24], a species that does not excrete Mus m 1 in the urine (Pavel Stopka, personal communication). This may explain why Mus m 1 was not detected in some of the samples from homes where mice had been reported. It is also possible that ‘mice’ sightings could in fact be juvenile brown rats, since non-experts sometimes have difficulty distinguishing between them. However, in the cases where mouse sightings were reported but no mouse allergen detected, no rat allergens were detected either. 

### 4.5. Pets in the Home

Of the participants who owned pets, more than half owned a cat and less than 30% owned a dog. In the univariate analysis the presence of a cat in the house was significantly associated with decreased odds of detectable mouse allergen in the house but was not significant in the multivariate analysis when other risk factors were included. Matsui et al. [8] also found an association between the presence of a cat in the house and decreased odds of detectable mouse allergen levels. It is possible that mice feel threatened by the presence of a cat in the house and stay away. However, some previous studies have shown that having pets in the house could be a risk factor for the presence of mouse allergen [9,18].

### 4.6. Exposure to Mouse Allergen and Asthma

No significant association was found between self-reported asthma and detectable mouse or rat allergens in the home. This is in contrast with findings in the USA, where mouse and rat allergen exposure in the home has been linked to mouse sensitization and asthma exacerbation among inner-city children [1,2,23]. Cleaning interventions and the implementation of integrated pest management (IPM) can lead to significant reductions in mouse allergen levels in homes [22]. However, reducing exposure to allergens does not always lead to a reduction in symptoms [25]. It is likely that the limited scale of our pilot compared to the larger observational studies contributed to the lack of an association.

### 4.7. Strengths and Limitations of this Study

The majority of homes sampled for allergens were concentrated around a city in the centre of the Netherlands. It is possible that a wider study including a larger number of homes would produce other associations. The survey relied on self-reported data, which may introduce some error into the analysis. However, participants were asked about building characteristics and cleaning routines in their own home, which makes it unlikely that they could be mistaken. Since rodent allergens were not specifically mentioned during recruitment in order to avoid selection bias, the frequency and levels of rodent allergens measured should be generalisable to other homes in the Netherlands.

## 5. Conclusions

The method of acquiring dustfall samples by sending out EDCs to the public is suitable and convenient, although if participants are recruited by flyering, the response rate is low. Mouse allergen was detected in almost half of the homes sampled and rat allergen in very few. Respondents who reported mouse sightings were more likely to have detectable mouse allergen in the home than respondents who reported no mouse sightings. Proximity to a green area and ventilation through an open window were the main predictors for detectable mouse allergen. Living close to open water and the use of furniture polish were protective.

## Figures and Tables

**Table 1:** Demographics of the population included in this study (n = 80).

Demographics of the Population Included in Study	Frequency (n = 80)	%	Utrecht (n = 63)	%	Other (n = 17)	%
**Gender**						
Male	24	30	21	33.3	3	17.6
Female	56	70	42	66.7	14	82.4
**Age**						
Between 20 and 40	20	25	13	20.6	7	41.2
Between 41 and 65	46	57.5	36	57.1	10	58.8
Over 65	14	17.5	14	22.2	0	0
**Education**						
Lower education	13	16.3	8	12.7	5	29.4
Higher education	67	83.3	55	87.3	12	70.6
**Smoke indoors (sometimes) (two missing values)**						
Sometimes	7	9	4	6.6	3	17.6
Participants that suffered from asthma	9	11.25	7	11.1	2	11.8
Pets at home (yes) (one missing value)	39	49.5	28	45.2	11	64.7
Cat	21	26.25	15	24.2	6	35.3
Dog	11	13.75	8	12.9	3	17.6
Mouse	0	0	0	0	0	0
Rat	1	1.25	0	0	1	5.9
**Sightings**						
Cockroaches sightings (yes)	0	0	0	0	0	0
Mouse sightings at home (yes)	32	40	26	41.3	6	35.3
Rat sightings at home (yes)	8	10	2	3.2	6	35.3
Detectable mouse allergens in the house	38	47.5	27	42.9	11	64.7
Detectable rat allergens in the house	3	3.8	1	1.6	2	11.8

**Table 2:** Household characteristics and reported cleaning habits of participants in this study (n = 80).

Household Characteristics and Reported Cleaning Habits	Frequency (n = 80)	%
**Distance from home to green area (m)**		
Less than 250	47	58.7
More than 250	33	41.3
**Distance from home to surface water (m)**		
Less than 250	45	56.3
More than 250	35	43.7
Private garden (yes)	68	85
**Age of the building**		
Before 1920	28	35
Between 1920–1960	32	40
After 1960	20	25
**Type of dwelling**		
Terraced house	51	63.7
Other	29	36.3
**Floor of the living room**		
Below ground or ground floor	63	78.7
First floor or higher	17	21.3
**Type of floor in the living room**		
Smooth (tiles, parquet, laminate)	61	76.3
Other	19	23.7
Insulated living room floor (yes)	41	51.2
Double glazed windows (yes)	65	81.3
**Ventilation**		
Through a ventilation grid (yes)	28	35
Through an open window (yes)	41	51.2
Through an open exterior door (yes)	50	62.5
Mechanical ventilation system (yes)	26	32.5
**Household conditions**		
Water damage or leakage (yes)	7	8.8
Mildew or mould spots (yes)	16	20
Smell of mildew or mould (yes)	9	11.3
**Usage of cleaning agents (sometimes)**		
Bleach/chlorine	60	75
Ammonia	18	22.5
Acids, liquid descaler	45	56.3
Solvents	35	43.8
Furniture polish	9	11.3
Cleaning spray	65	81.3
Degreasing sprays	52	65
Polishes or wax for the floor or furniture	48	60
Multipurpose cleaner	50	62.5
**High frequency of cleaning habits (more than 1x per week)**		
Cleaning in general	49	61.2
Vacuuming	56	70
Sweeping	35	43.8
Dusting	30	37.5
Wet cleaning of the floor	21	26.2

**Table 3:** Logistic regression for predictors of Mus m 1 allergen detection in 80 Dutch households.

	Univariate Analysis				Multivariate Analysis (All Cases, n = 80)				Multivariate Analysis (Cases in Utrecht only, n = 63)			
Variables *	Exp(B) OR	95% C.I. for EXP(B)		Sig.	Exp(B) OR	95% C.I. for EXP(B)		Sig.	Exp(B) OR	95% C.I. for EXP(B)		Sig.
		Lower	Upper			lower	Upper			lower	upper	
**Less than 250 m from green area**	2.17	0.87	5.26	0.097	**5.56**	**1.59**	**20.00**	**0.008**	**7.25**	**1.41**	**37.04**	**0.018**
**Less than 250 m from surface water**	0.50	0.20	1.22	0.130	**0.24**	**0.07**	**0.84**	**0.025**				
**House built before 1920**	0.77	0.24	2.46	0.658					**6.11**	**1.20**	**31.17**	**0.03**
House built between 1920–1960	0.35	0.11	1.11	0.074								
House built after 1960	1											
House has a private garden	0.89	0.26	3.03	0.851					7.00	0.68	71.66	0.10
**Living room floor is insulated**	2.04	0.84	4.99	0.116	**3.28**	**1.04**	**10.36**	**0.042**	**13.25**	**2.52**	**69.57**	**0.002**
**Living room is ventilated by an open window**	**3.13**	**1.25**	**7.80**	**0.015**	**6.10**	**1.89**	**19.71**	**0.002**	**7.65**	**1.51**	**38.91**	**0.014**
Smell of mildew/mould in the last 12 months	1.44	0.36	5.81	0.609								
**Furniture polish used**	0.51	0.12	2.22	0.373	**0.11**	**0.01**	**0.81**	**0.03**				
Multipurpose cleaner used	0.90	0.05	14.95	0.943								
Degreasing spray used	1.07	0.43	2.68	0.888								
Solvents used	0.88	0.36	2.13	0.778								
Dusting at least once a week	1.17	0.47	2.91	0.729								
Vacuuming at least once a week	1.40	0.53	3.68	0.495								
Wet cleaning of floor at least once a week	2.21	0.80	6.14	0.128								
**Mouse sightings in the last 12 months**	2.23	0.90	5.56	0.085	2.90	0.888	9.46	0.078	**4.78**	**1.14**	**20.06**	**0.033**
**Cat as pet**	**0.25**	**0.08**	**0.79**	**0.017**								

* Variables with *p* < 0.05 are in **bold**.

**Table 4:** Linear regression for predictors of the level of Mus m 1 allergen in 80 Dutch households.

	Univariate Analysis				Multivariate Analysis (All Cases, n = 80)				Multivariate Analysis (Cases in Utrecht only, n = 63)			
Variables *	GMR	95.0% Confidence Interval for GMR		Sig.	GMR	95.0% Confidence Interval for GMR		Sig.	GMR	95.0% Confidence Interval for GMR		Sig.
		Lower	Upper			Lower	Upper			Lower	Upper	
**Less than 250 m from green area**	1.14	0.79	1.75	0.430	**1.43**	**1.20**	**1.93**	**0.001**	**1.65**	**1.38**	**2.32**	**0**
**Less than 250 m from surface water**	0.89	0.60	1.27	0.476	**0.73**	**0.54**	**0.88**	**0.003**	**0.77**	**0.54**	**0.95**	**0.021**
House built before 1920	1.06	0.76	1.49	0.692								
House built between 1920–1960	0.85	0.60	1.15	0.256								
House built after 1960 (= reference)	1.00											
House has a private garden	0.84	0.46	1.26	0.278								
**Living room floor is insulated**	1.03	0.70	1.52	0.850					**1.23**	**1.00**	**1.65**	**0.048**
**Living room is ventilated by an open window**	0.90	0.59	1.31	0.536	**1.28**	**1.06**	**1.67**	**0.014**				
**Smell of mildew/mould in the last 12 months**	0.89	0.47	1.43	0.481	0.81	0.46	1.01	0.053	**0.78**	**0.34**	**0.91**	**0.021**
**Furniture polish used**	0.99	0.49	1.97	0.961	**0.77**	**0.41**	**0.90**	**0.013**				
**Multipurpose cleaner used**	0.73	0.11	1.03	0.057	**0.81**	**0.23**	**0.97**	**0.042**				
Degreasing spray used	0.75	0.49	1.04	0.076	0.82	0.63	1.00	0.053				
**Solvents used**	0.82	0.55	1.16	0.222					**0.76**	**0.56**	**0.93**	**0.013**
**Dusting at least once a week**	**0.64**	**0.43**	**0.86**	**0.006**					**0.68**	**0.47**	**0.84**	**0.003**
Vacuuming at least once a week	0.93	0.59	1.4	0.669					1.22	0.95	1.75	0.1
Wet cleaning of floor at least once a week	0.74	0.48	1.03	0.072								
Mouse sightings in the last 12 months	1.11	0.77	1.63	0.552	1.20	0.96	1.55	0.09	1.22	0.98	1.65	0.071
Cat as pet	1.05	0.61	1.9	0.787								

* Variables with *p* < 0.05 are in **bold**.

## Data Availability

The data supporting the findings of this study are available from the corresponding author upon reasonable request.

## Supplementary Materials

The following are available online at https://www.mdpi.com/1660-4601/16/19/3736/s1, Figure S1: Map of the Netherlands showing the geographical distribution of homes sampled in this study (n = 80). File S1: Questionnaire.
