# Supplementary material for: Airborne Rodent Allergen Levels in Dutch Households: A Pilot Study
Source: Int J Environ Res Public Health. 2019 Oct 4;16(19):3736. doi: 10.3390/ijerph16193736 (PMC6801909; doi:10.3390/ijerph16193736)
Supplement: Supplementary file 1 [file ijerph-16-03736-s001.zip › IJERPH_Questionnaire_allergens_English_def.pdf]

Universiteit Utrecht

QUESTIONNAIRE FOR THE STUDY OF ALLERGENS IN DUTCH HOUSEHOLDS

Explanation of the questionnaire

This is the questionnaire belonging to the study ‘Allergens in Dutch households’ and contains questions about your home, household habits and your health.

Most questions can be answered by placing a cross next to the appropriate answer. In some cases you may find it difficult to make a choice between the answers. For the purposes of this study it is best if you do make a choice and in that case you should choose the answer that is most appropriate. In several places you will be asked to fill in an answer, in which case you should write your answer in the space provided.

Please complete this questionnaire and to return it to us in the envelope with the dust collection cloth.

To protect your privacy, your details will be entered into the computer separate from your name and address and only identifiable as a number.

If you have any questions please contact:

Ms S.A. Burt, PhD

030-253.5350

s.a.burt@uu.nl

<u>This page will be removed on arrival and stored separately from the answers</u>

I confirm that I wish to take part in the study about the presence of allergens in Dutch households.

PERSONAL INFORMATION

Mr / Ms *(delete as appropriate)*

Initials: ……..……….. Surname (family name): …………………………….……………………..

Address.

(street + house number) ……………………………………………….…………………………

(postcode + town) …………………………………………….……………………………

Telephone: (optional) ……………………………………………….…………………………

Email: (optional) ……………………………………….……………………………

Signature …………………………………………..……………………………….

❑ I wish to receive an email with a summary of the results of the study.

A. General questions

A.1 Are you male or female?

- ❍ Male
- ❍ Female

A.2 What is your age?

|______| *years*

A.3 In which country were you born?

- ❍ Netherlands
- ❍ Other country, namely ……………………………………………………………

A.4 Apart from you, how many other people are there in your household?
(more than 1 answer is possible)

- ❑ I live alone
- ❑ I live with a partner
- ❑ My parents or other adult live(s) with me: |______| (*number*)
- ❑ Children 18 years or older: |______| (*number*)
- ❑ Children 13 - 17 years: |______| (*number*)
- ❑ Children 0 - 12 years: |______| (*number*)

A.5 What is the highest level of education you have achieved?

- ❍ No diploma's
- ❍ Primary school
- ❍ *Lager / voorbereidend beroepsonderwijs (LTS, LEAO, LHNO, VMBO)*
- ❍ *Middelbaar voortgezet onderwijs (MAVO, MULO, MBO-kort, VMBO-t)*
- ❍ *Middelbaar beroepsonderwijs (MBO-lang, MTS, MEAO, BOL, BBS, INAS)*
- ❍ *Middelbaar hoger onderwijs (HAVO, VWO, Atheneum, Gymnasium, HBS, MMS)*
- ❍ *Hoger beroepsonderwijs (HBO, HTS, HEAO)*
- ❍ University

B. Health

B.1 How would you describe your health **at the moment**?

❍ Bad ❍ Fair ❍ Reasonable ❍ Good ❍ Excellent

B.2 Have you ever had asthma?

❍ Yes ❍ No *(If no, continue from question B.8)*

If yes, ...

B.3 Has the asthma been confirmed by a doctor?

❍ No

❍ Yes, that was in |__|__|__|__| (*year*)

B.4 How old were you when you had your first asthma attack?

I was then |______| years old

B.5 Have you had an asthma attack **in the last 12 months?**

❍ Yes ❍ No *(If no, continue from question B.7)*

If yes, ...

B.6 How many asthma attacks have you had **in the last 12 months?**

|______| attacks

B.7 Do you use asthma medicine at the moment?
(for example inhalers, aerosols, tablets)

❍ Yes ❍ No

B.8 Are you, or have you ever been over-sensitive or allergic to something?

❍ Yes ❍ No *(If no, continue with Part C of the questionnaire)*

If yes, ...

B.9 Please indicate with a cross which things you are over-sensitive or allergic to.
Please also indicate which symptoms you have.
(several answers possible)

Over-sensitive or allergic to:		*If yes, symptoms of over-sensitivity or allergy:* Sneeze or runny nose	Wheezing	Itchy or red skin	Itchy or teary eyes
**A.** House dust	❍ No ❍ Yes →	❑	❑	❑	❑
**B.** Food	❍ No ❍ Yes →	❑	❑	❑	❑
**C.** Animals	❍ No ❍ Yes →	❑	❑	❑	❑
**D.** Plants or pollen	❍ No ❍ Yes →	❑	❑	❑	❑
❑ Other, namely		❑	❑	❑	❑

If you are over-sensitive or allergic to animals …

B.10 Which animal(s) are you allergic to?
(*more than one answer is possible)*

❑ Cat ❑ Dog ❑ Rabbit ❑ Guinea pig or Hamster

❑ Horse ❑ Bird ❑ Mouse ❑ Rat

❑ Other, namely ....

C. Your home

C.1 When did you move into your present home?

|__|__| - |__|__| - |__|__|__|__| (*dd – mm – yyyy*)

day month year

C.2 When was your home built?

- ❍ before 1920
- ❍ between 1920 and 1960
- ❍ between 1960 and 1980
- ❍ between 1980 and 2000
- ❍ after 2000
- ❍ I don't know

C.3 What type of house is your present home?

(only choose one answer: choose the answer that fits your home best)

- ❍ detached house
- ❍ semi-detached
- ❍ terraced
- ❍ flat
- ❍ maisonette
- ❍ ground floor duplex
- ❍ upper floor duplex
- ❍ other, namely. . . . .

C.4 Do you have a garden next to your home?

- ❍ yes
- ❍ no

C.5 How far from your home do you estimate the nearest **green area (park, recreation ground**) to be?

- ❍ less than 100 metres
- ❍ between 100 - 250 metres
- ❍ between 250 - 500 metres
- ❍ between 500 - 1000 metres
- ❍ more than 1000 metres

C.6 How far from your home do you estimate the nearest water course **(canal, lake, river**) to be?

- ❍ less than 100 metres
- ❍ between 100 - 250 metres
- ❍ between 250 - 500 metres
- ❍ between 500 - 1000 metres
- ❍ more than 1000 metres

C.7 Please answer the following questions for your living room (lounge)

		**Living room**
On which floor is this room?		
Lower than the ground floor	(1)	❍
On the ground floor	(2)	❍
1st floor	(3)	❍
2nd floor	(4)	❍
3rd floor	(5)	❍
Higher than 3rd floor	(6)	❍
Is the floor insulated?		
yes	(1)	❍
no	(2)	❍
I don't know	(3)	❍
Are the windows double glazed?		
yes	(1)	❍
no	(2)	❍
I don't know	(3)	❍
Wat type of floor covering is in the living room?		
hard (vinyl/linoleum/tiles/wood/laminated)	(1)	❍
wall to wall carpeting (synthetic/wool)	(2)	❍
hard floor with loose rug(s)	(3)	❍

D. Ventilation

D.1 How do you normally ventilate the living room?

(several answers are possible)

❑ by means of a ventilation grille

❑ by means of an open window

❑ by opening an external door

D.2 Does your home have a mechanical ventilation system?
(*continuous extraction of air from the kitchen, toilet and bathroom*)

❍ yes

❍ no

❍ I don't know

D.3 Have you had water damage or a leak in the house **in the last 12 months?**

❍ yes ❍ no

D.4 Have you seen patches of mould or mildew on the wall or ceiling **in the last 12 months?**

❍ yes ❍ no

D.5 Have you smelt mould or damp in the house **in the last 12 months?**

❍ yes ❍ no

D.6 Have you seen cockroaches in the house **in the last 12 months?**

❍ yes ❍ no

D.7 Have you seen mice in the house **in the last 12 months?**

❍ yes ❍ no

D.8 Have you seen rats in the house **in the last 12 months?**

❍ yes ❍ no

D.9 How often have you or others used the following cleaning agents in the last 12 months?
(*Please place one cross for each cleaning agent*)

	Never	Less than 1x per week	1-3x per week	4-7x per week
Bleach	❍	❍	❍	❍
Ammonia	❍	❍	❍	❍
Acids or descaler	❍	❍	❍	❍
Solvents (or stain removers)	❍	❍	❍	❍
Furniture polish	❍	❍	❍	❍
Glass cleaning spray (for windows and mirrors)	❍	❍	❍	❍
De-greasing sprays or oven cleaner	❍	❍	❍	❍
Polish or wax for the floor or furniture	❍	❍	❍	❍
General purpose cleaning liquid	❍	❍	❍	❍

D.10 How often do you or other people normally clean the living room?
(Please place one cross per method of cleaning)

	Never	Less than 1x per week	1-3x per week	4-7x per week
General cleaning	❍	❍	❍	❍
Vacuuming	❍	❍	❍	❍
Sweeping	❍	❍	❍	❍
Dusting	❍	❍	❍	❍
Wet cleaning of the floor (e.g. with mop)	❍	❍	❍	❍

D.11 Do people smoke **inside** your home?

❍ Seldom or never ❍ Sometimes ❍ Regularly ❍ (Almost) daily

E. Pets

E.1 Have you kept pets in the house **in the past 12 months?**

❍ yes ❍ no

If yes, ...

E.2 Which sort of pets have you kept in the house **in the last 12 months?**
(*Please indicate what is relevant for each type of pet below*)

Type of pet …	*No*	*Yes*
**1.** Cat	❍	❍
**2.** Dog	❍	❍
**3.** Bird	❍	❍
**4.** Rabbit	❍	❍
**5.** Guinea pig	❍	❍
**6.** Hamster	❍	❍
**7.** Mouse	❍	❍
**8.** Rat	❍	❍
**9.** Fish	❍	❍
**10.** Tortoise/turtle	❍	❍

----- END -----
