# Supplementary figures and images for: Airborne Rodent Allergen Levels in Dutch Households: A Pilot Study
Source: Int J Environ Res Public Health. 2019 Oct 4;16(19):3736. doi: 10.3390/ijerph16193736 (PMC6801909; doi:10.3390/ijerph16193736)

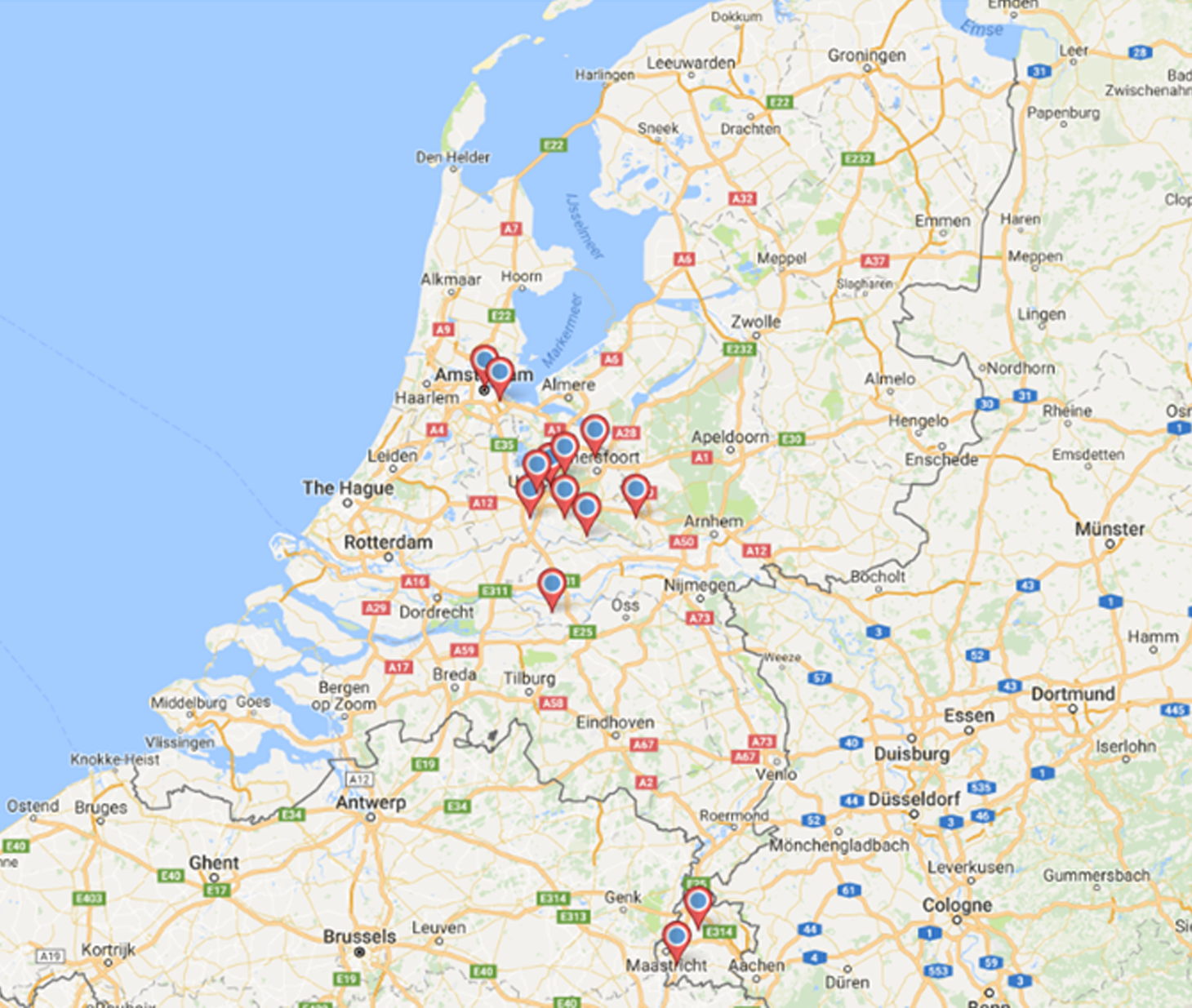

Supplement: Supplementary file 1 [file ijerph-16-03736-s001.zip › IJERPH_Allergens_Suppl_Figure S1.tif]
